# Biomaterial Scaffolds in Regenerative Therapy of the Central Nervous System

**DOI:** 10.1155/2018/7848901

**Published:** 2018-04-01

**Authors:** Yanchao Wang, Hong Tan, Xuhui Hui

**Affiliations:** ^1^Department of Neurosurgery, West China Hospital, Sichuan University, Chengdu, Sichuan, China; ^2^College of Polymer Science and Engineering, State Key Laboratory of Polymer Materials Engineering, Sichuan University, Chengdu, Sichuan, China

## Abstract

The central nervous system (CNS) is the most important section of the nervous system as it regulates the function of various organs. Injury to the CNS causes impairment of neurological functions in corresponding sites and further leads to long-term patient disability. CNS regeneration is difficult because of its poor response to treatment and, to date, no effective therapies have been found to rectify CNS injuries. Biomaterial scaffolds have been applied with promising results in regeneration medicine. They also show great potential in CNS regeneration for tissue repair and functional recovery. Biomaterial scaffolds are applied in CNS regeneration predominantly as hydrogels and biodegradable scaffolds. They can act as cellular supportive scaffolds to facilitate cell infiltration and proliferation. They can also be combined with cell therapy to repair CNS injury. This review discusses the categories and progression of the biomaterial scaffolds that are applied in CNS regeneration.

## 1. Introduction

The central nervous system (CNS), which comprises the brain and spinal cord, is the most important and complex part of the nervous system. Two of the most common causes of injury to the CNS are trauma [1] and hemorrhage [2]. For example, approximately 1.5 million individuals in the USA suffer traumatic CNS injury annually, which includes spinal cord injury (SCI) and traumatic brain injury (TBI) [3, 4]. Injury to the CNS causes significant mortality and morbidity, which results in a heavy economic burden on society. It is reported that, for 2010, the economic burden of TBI on the US economy was approximately $76.5 billion [4, 5].

Pathologically, CNS injury can directly result in the death of parenchymal cells in damaged tissue [6]. CNS injury can also cause secondary injury, such as hemorrhage, edema, and cell apoptosis due to the persisted inflammation caused by accumulated immune cells after injury [7]. In the pathological tissue, both neutrophils and macrophages adopt an inflammatory phenotype and release soluble factors, including cytokines, proteolytic enzymes, and oxidative metabolites, that exacerbate injury [8]. Leakage can also occur across the blood-brain barrier (BBB), aggravating the inflammation and damaging tissues [9–11]. The primary CNS injury in combination with its subsequent side effects may cause long-term disease and mortality [12–14]. Instinctive CNS repair processes, including accumulation of endogenous stem cells, inflammatory cells, and astrocytes; secretion of chemokines; and formation of glia scar, occur spontaneously to mitigate CNS injury [14, 15]. These mechanisms can partially rescue the residual cells and repair injured tissues. However, the endogenous repair mechanisms modify the components of the extracellular matrix (ECM) of lesions and subsequently cause further ECM degradation and remodeling [16, 17]. The chemokines (e.g., CCL-2, IL-6, and TNF-*α*) secreted by inflammatory cells can also aggravate local inflammatory reactions [18, 19]. These microenvironmental changes cause failure of stem cells to differentiate into nerve cells and also impede axon regrowth by survival neurons [7, 16]. Further, the glia scars formed by reactive astrocytes, microglia cells, and deposited chondroitin sulphate proteoglycans (GMPGs) separate the lesion from the surrounding tissue and hamper CNS regeneration [15, 20, 21].

Recovery from CNS injury requires rescuing of the surviving cells and axons, repairing damaged tissue, regeneration of severed axons, reconstruction of the connection between the nervous process and soma, and rehabilitation of the impaired neural functions. In recent years, with developments in stem cell biology, cell therapies have been introduced into CNS regeneration [22, 23]. Numerous studies have reported that transplantation of fetal tissue/stem cells into damaged CNS tissues can give favorable results, such as axonal regrowth and regeneration of neurons [24–26]. However, cell therapies have proven inadequate for certain CNS injuries because when a lesion is too wide cell therapy alone cannot repair it; extra physical support is needed to enable engraftment of transplanted cells and cytoarchitecture restoration [6, 24, 27, 28]. Consequently, there are currently no effective therapies for CNS injury.

Biomaterial scaffolds have been studied in tissue regeneration for decades. They have been utilized for regeneration of soft tissue, cartilage, bone, and the peripheral nerve system (PNS) with favorable results [29–32]. Biomaterial scaffolds have a three-dimensional (3D) architecture and are designed to replicate the interaction between cells and their native extracellular matrix (ECM) microenvironments [33, 34]. They can also function as a reservoir for controlled therapeutic molecule delivery or cell transplantation [35]. Recently, numerous studies have revealed that incorporation of biomaterial scaffolds has promoted CNS tissue regeneration in repair of both SCI and TBI [36, 37]. It has been shown that biomaterial scaffolds can repair CNS injury, alter the microenvironment of lesions, and promote the recovery of neural function [38, 39]. Thus, it is clear that biomaterial scaffolds are playing an increasingly important role in CNS regeneration. This review discusses the categories of biomaterial scaffolds that are applied in CNS regeneration as well as their effects.

## 2. Categories of Biomaterial Scaffolds Applied in CNS Regeneration

Biomaterial scaffolds are used in effort to provide specific microenvironmental cues in 3D controlled fashion to enhance cell survival, infiltration, and differentiation. Since the revelation by David and Aguayo [40] of the significance of microenvironments in CNS repair, it has been asserted that modulating hostile CNS microenvironments can improve recovery from CNS injury. Biomaterial scaffolds and biological scaffolds are the two main scaffolds utilized in CNS regeneration. Both types of scaffolds have a 3D topological structure that can closely mimic the native extracellular matrix (ECM). However, whereas biomaterial scaffolds are composed of synthesized polymers or purified natural polymers, biological scaffolds are usually in the form of decellularized mammalian tissue [38, 41–43]. Further, biomaterial scaffolds are superior to biological scaffolds in key parameters such as architecture, pattern, biocompatibility, porosity, and stiffness, and their degradation rate can be modulated more easily and precisely [44].

**Figure 1:**
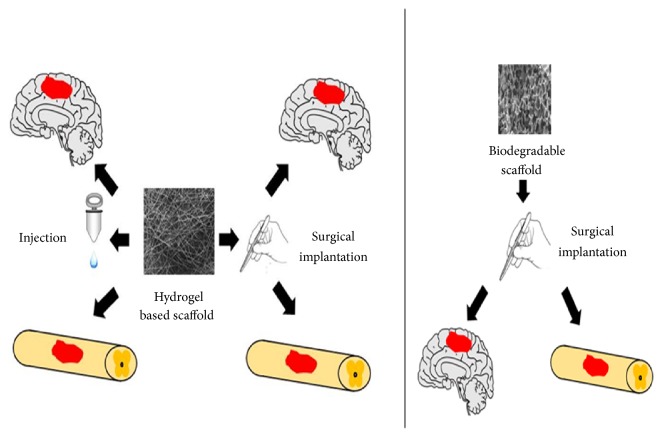
Application of biomaterial scaffolds in regeneration of central nervous system.

Biomaterial scaffolds that serve as temporary ECM provide a niche for cell infiltration and differentiation. They not only support the surrounding neural tissue but also act as a substrate for cell growth, neurite formation, and axon regeneration. They can also carry bioactive molecules that can create a relatively stable, permeable, and nutritious environment for regeneration [45–48]. Moreover, biomaterial scaffolds can also be combined with cell therapies to form “live” scaffolds. The combination of cell therapies and biomaterial scaffolds can provide physical support for transplanted cell engraftment and isolate the implanted cell from the host tissue to provide an independent microenvironment for cell differentiation and proliferation (Figure 1) [49].

Based on required structure and physical and biological properties of prospective tissue construct applied in CNS injury, the biomaterial scaffolds utilized in CNS regeneration can be further classified into hydrogels and biodegradable scaffolds. In this section, we introduce the categories of hydrogels and biodegradable scaffolds utilized in CNS regeneration.

### 2.1. Hydrogels

Hydrogels are an attractive scaffold substrata owing to their high water content and porous inner structure, which makes them soft and flexible and minimizes tissue damage [50–53]. Their 3D inner structure extends the surface that makes contact with infiltrated cells and expands their volume. Numerous studies have indicated that hydrogels can promote cell adhesion, axon regeneration, and myelination in CNS injury both* in vitro* and* in vivo* [54–56].

Hydrogels can be classified into polymeric covalently cross-linked hydrogels and self-assembled hydrogels according to the forming mechanism [24, 51]. In polymeric covalently cross-linked hydrogels, monomer units are linked by covalent forces, which makes hydrogels more stable in alteration of environment parameters such as pH and temperature [30]. Because they are cross-linked through covalent forces, polymeric covalently cross-linked hydrogels often appear as having an aligned inner structure. High percentage of covalent bonds between inner polymer molecules makes covalently cross-linked hydrogels less deformable but stiffer. Thus, they are usually implanted surgically [57, 58]. In self-assembled hydrogels, monomer units are organized by internal noncovalent forces, which results in them having soft and deformable mechanical characteristics. The noncovalent forces also cause self-assembled hydrogels to have randomly oriented inner structures. Self-assembled hydrogels self-assemble into hydrogels through the environmental PH or temperature changes. Thus, they can be easily injected into lesions [59, 60].

Hydrogel forming polymeric materials are classified as either natural materials or synthetic materials [61]. Natural materials are often used to produce polymeric covalently cross-linked hydrogels. They are obtained from natural resources such as hyaluronic acid from roster comb [62], fibroin [63, 64], chitosan [65], collagen from the epithelial tissue of calf [66, 67], and alginate from seaweed algae [68, 69]. Further, they are easy to acquire, contain specific molecules for cell adhesion, are biodegradable, and are highly biocompatible [70, 71]. However, natural materials also have insufficiencies such as variations between batches, which makes it hard to control the homogeneity of resulting scaffolds. In addition, the natural sources from which they are derived may contain immune reaction-causing pathogens [72].

Ethyleneglycol monomethacrylate (HEMA) and ethylene dimethacrylate (EDMA) are the first materials reportedly used to synthesize polymeric covalently cross-linked hydrogels [73, 74]. Nowadays, the hydrogels created from synthetic materials hydrogels that are widely utilized in CNS are usually synthesized from polyethylene glycol (PEG) [75], poly-N-(2-hydroxyethyl) methacrylamide (PHEMA), or poly-N-(2-hydroxypropyl) methacrylamide (PHPMA) [76–78]. Self-assembling peptides (SAPs) are the main type of self-assembled hydrogels. They have short, repeating units of amino acids and altered polar and nonpolar residues that enable them to form double-*β* sheet structures when dissolved in water [79, 80]. The first reported SAP was EAK16-II [81]. Subsequently, other derivatives SAPs such as RADA16 and KLDL12 family were developed as 3D scaffolds for cells [82–85]. These scaffolds can mimic the structure of ECM and functional sequences such as RGD can be added to their self-assembling sequence to improve cell adhesion, proliferation, differentiation, and maturation [86–88]. Peptide amphiphile molecules (PAs) are another important class of SAPs. These SAPs can change the interior array of hydrogels and improve their regeneration effect in the nervous system [89, 90]. Moreover, SAPs hydrogels can also carry function molecules such as homing peptides and neurotrophic factors to promote regeneration (Table 1) [88].

### 2.2. Biodegradable Scaffolds

Biodegradable scaffolds are biomaterials characterized by biodegradability and 3D inner architecture. Their 3D porous structures are fabricated through methods such as electrospinning, freeze-drying, microfluidic fabrication, water emulsion, thermoforming, and 3D printing [91–93]. The scaffold should be progressively replaced by the regenerating tissue, in order to last long enough to permit cell infiltration and support axon regrowth; moreover, their degraded product must be nontoxic [94]. Degradation of biodegradable scaffolds can occur by hydrolysis and enzymatic degradation, or as a result of mechanical and oxidative stress in vivo [95]. Their degradation rate can be regulated through tuning degree of acetylation, stiffness of scaffold, and changing the length of hydrolytically degradable units within the polymer crosslink [24]. Biodegradable scaffolds exhibit high biodegradability and biocompatibility that minimize their side effects on tissues and attenuate inflammation in lesions. Further, their mechanical property, porosity, shape, and conduits alignment can be easily adjusted through control of rate of cross-linking or concentration of reactants and flow rate of extruded substrata in biofabrication [96]. It has been asserted that biodegradable scaffolds are suitable for utilization in nervous tissue as they can mimic the microstructure and elastic modulus of the ECM of nerve tissues [6, 97]. Moreover, biodegradable scaffolds can carry ECM proteins, growth factors, or stem cells to generate functional scaffolds [98, 99]. Biodegradable scaffolds are desirable constructs to utilize in vivo as well as in vitro applications.

Biodegradable scaffolds can also be synthesized from natural materials or synthetic materials. The natural materials often used for this purpose include collagen [100], fibroin protein (e.g., silk fibroin) [101], chitosan, and hyaluronic acid [51, 102]. The synthetic materials that have been used to synthesize biodegradable scaffolds include poly *ε*-caprolactone (PCL) [103], poly L-lactic acid (PLA), and polyurethane [104, 105]. However, materials such as PCL are hydrophobic, which may lead to poor cell interactivity and further impede cell adhesion and proliferation [106]. To solve this problem, copolymer biodegradable scaffolds have been developed as a compromise method. This method introduces two or more different species into the polymer chain of macromolecules to promote hydrophilicity in scaffolds. The poly D,L-lactide-*co*-glycolic acid (PLGA) [107] and poly *ε*-caprolactone-*co*-ethyl ethylene phosphate (PCLEEP) are common copolymers that are utilized in nerve system regeneration [108]. Synthetic materials can also be combined with other synthetic materials or natural materials to create copolymers, such as PCL-PLGA scaffolds, which also combines the properties of each material and intensifies the regeneration capacity of the scaffolds (Table 2) [107]. For synthetic materials scaffolds, to achieve a specific degradation rate, oligopeptides that are sensitive to the enzymatic cleavage have been engineered into synthetic polymers. This results in the fact that hydrogels are specifically degraded by targeted enzymes involved in matrix remodeling such as matrix metalloproteases (MMPs), collagenases, and plasmin [24, 94].

Both hydrogels and biodegradable scaffold are important biomaterial scaffolds utilized in CNS regeneration. They can serve as temporary ECM to provide a niche for cell infiltration and differentiation. For future study, the choice of suitable materials for scaffold synthesis and techniques for fabricated 3D structure nontoxically should be important issues in scaffold synthesis. Besides in vivo interaction between the ECMs and scaffolds and the mechanisms of degradation still need further study.

## 3. Biomaterial Scaffolds in Spinal Cord Regeneration

Spinal cord injury (SCI) is characterized by long-term paralysis and sensory disturbances. SCI patients often lose the ability to work and require lifelong care [109]. Although much effort has been made by clinicians and scientists to cure this disability, the outcome for SCI patients is still unsatisfactory. In this section, we focus on the properties and mechanisms of non-cell therapy biomaterial scaffolds that have been applied in the treatment of SCI. Biomaterial scaffolds that are combined with cell therapy and applied in SCI are discussed individually in Section 5.

### 3.1. Application of Hydrogel in SCI

Natural polymer-derived hydrogels were first applied to SCI in 1995, when Joosten et al. used collagen hydrogels in experimental SCI model. They compared two methods of collagen hydrogels preparation, as either a fluid or preformed solid gel, in a rat SCI model. Their results showed that even though both scaffolds can reduce the gliotic response, only fluid collagen gel can induce regeneration of damaged axons [110]. Their study also resulted in a new solution for SCI. Subsequently, the effects of hydrogels made from other natural materials in the treatment of SCI have been intensively studied. It has been found that fibrin hydrogels improve tissue repair and axon regrowth [111], chitosan hydrogels promote tissue repair and neuroprotection in the SCI model, and alginate hydrogels promote axonal regrowth and elongation [112]. With the development of synthetic hydrogel techniques, raw natural material hydrogels have been designed to carry drugs and neurotrophic factors to enhance their SCI reparative effect. For example, Furuya et al. [113] injected gelatin hydrogel (GH) containing basic fibroblast growth factor (bFGF) into a rat SCI model. The bFGF-incorporated GH showed better performance in alleviating mechanical allodynia following SCI. Further, drugs such as methylprednisolone are also able to enhance axonal regeneration and reduce inflammation [114, 115]. It has been stated that excessive Ca^2+^ can hamper neurite formation and axon regrowth. To overcome this problem, McKay et al. [116] developed alginate/chitosan/genipin hydrogels, which have a high sensitivity to Ca^2+^ composites. The developed hydrogels exhibited excellent ability to regulate astrocyte behavior and prevent Ca^2+^-related secondary neuron damage during acute SCI.

Hydrogels made from natural materials can also deliver specific antibodies or drugs to block receptors that impede regeneration after SCI. Nogo is a myelin-associated inhibitor (MAI) that can limit axon growth and benumb functional neuronal circuits. Wen et al. developed hyaluronic acid (HA) hydrogels that blend with the anti-Nogo receptor antibody (antiNgR). Hydrogels have also been combined with PLGA microspheres containing brain-derived neurotrophic factor (BDNF) and vascular endothelial growth factor (VEGF). The hydrogels were implanted into the rat SCI model and, after a few weeks, angiogenesis and axons regrowth were observed in hydrogels; the implanted rats also exhibited improved locomotor recovery [117]. These studies prove that hydrogels made from natural materials are effective in SCI treatment. They are highly biocompatible and contain a specific molecule for cell adhesion; their inner structure can mimic the extracellular matrix to provide an environment for cell proliferation.

Similar to natural hydrogels, hydrogels made from synthetic materials for the treatment of SCI can mimic the extracellular matrix to provide an environment for cell proliferation and adhesion. Further, hydrogel networks can serve as scaffolds that support regeneration until the materials are ultimately absorbed by the host. Hydrogels made from synthetic materials are more adjustable than hydrogels made from natural materials, as their key parameters can be easily controlled through modification. Poly(hydroxyethyl methacrylate) (PHEMA) was one of the earliest biomaterials utilized for tissue engineering scaffolds as they alleviate inflammation and promote axon regeneration after SCI [118]. Subsequently, biocompatible hydrogels such as polyethylene glycol (PEG) and poly-N-(2-hydroxypropyl) methacrylamide (PHPMA) hydrogels have been utilized in SCI treatment. Namba et al. [119] applied porous PEG hydrogels to SCI. They demonstrated that PEG hydrogels are simple and efficient and enable uniform seeding of neural cells throughout the entire porous scaffolds, thereby promoting axon regeneration. PHPMA hydrogels exhibit reduced macrophages/monocytes accumulation at the lesion border, and axons and myelin are both preserved in the rostral and caudal of the lesion [76]. Many aspects of hydrogels made from synthetic materials, such as phase, stiffness, biodegradability, and pattern, can also be modified to provide precise temporal control of the hydrogels and host cell interactions. For example, the hydrogels can be charged; positively charged hydrogels display higher cell infiltration and growth than negatively charged hydrogels [120].

Hydrogels made from synthetic materials can also act as a carrier to deliver growth factor to lesions and enhance their reparative effect. Chen et al. [121] incorporated basic fibroblast growth factor (bFGF) into hydroxyl ethyl methacrylate [2-(methacryloyloxy)ethyl] trimethylammonium chloride (HEMA-MOETACL) hydrogels and implanted them into the lesion of an SCI model. Their results showed that the hydrogels promoted both nerve tissue regeneration and functional recovery in the SCI model.

Adjunction of functional sequence is also a common method used to modify hydrogels. RGD [122], IKVAV [123], and laminin [124] are functional sequences that are often utilized to modify hydrogels. These functional sequences can enhance the treatment effects of scaffolds by promoting cell adhesion and proliferation in scaffolds. Woerly et al. [125] synthesized poly-N-(2-hydroxypropyl) methacrylamide (PHPMA) based hydrogels and demonstrated that they can promote axonal regeneration in an experimental SCI model. They further decorated PHPMA hydrogels with an RGD sequence and showed that the modified hydrogels can induce tissue ingrowth into the lesion cavity, and angiogenesis and axon regeneration are more effective in modified hydrogels.

SAPs and PAs are important synthetic polymers for producing self-assembling hydrogels. The self-assembling hydrogels are injectable and facilitate clinical application. Gou et al. [126] were the first to apply RADA16-I hydrogels to an experimental SCI model and prove that SAP hydrogels can promote SCI recovery. Cigognini et al. [127] further functionalized RADA16-I hydrogels with a bone marrow homing motif (BMHP1). To facilitate scaffold stability and expose more biomotifs, they inserted 4-glycine-spacer into the hydrogels. Their results indicated that RADA16-I hydrogels can increase cell infiltration, basement membrane deposition, and axon regeneration in SCI. Tysseling et al. [128] applied the functional sequence IKVAV to modified PA hydrogels and implanted them into a rat SCI model. Their results showed that, in contrast to randomized functional sequences, IKVAV PA hydrogels can improve histological and functional recovery. Their results also suggest that proper matching of functional sequence and hydrogels may be important in the synthesis of functional hydrogels.

Neuroinflammation develops within hours after SCI and TBI and can persist for months to years [11]. Delivering interventions following injury may be critical for regeneration and restraining lesion expansion [129]. Monocyte-derived macrophages are early responders to injury [130]. Both* in vitro* and* in vivo* evidences demonstrate that with specific stimulation macrophages can polarize towards functionally divergent subsets. Historically, polarized macrophages have been classified as classical (M1) macrophages, which promote inflammation, or as alternatively activated (M2) macrophages, which restrict inflammation and foster wound repair. Outside the CNS, M1 macrophages are quite rapidly (after about 1 week) replaced by M2 macrophages that successively infiltrate the lesion, where they largely contribute first to tissue repair and then remodeling via release of anti-inflammatory cytokines, stimulation of proliferation of fibroblasts and endothelial cells (angiogenesis), and production of ECM [131–134]. However, in traumatic SCI, this counterbalancing is impaired. The M2 macrophages are activated early, but disappear after about one week after lesion, while proinflammatory M1 macrophages persist indefinitely [135]. Similarly, in TBI, field alternation of M1 and M2 is also observed through numerous studies [136, 137]. Hydrogels made from both natural and synthetic materials are anti-inflammatory and alleviate gliosis after SCI, providing a favorable microenvironment for regeneration. Furthermore, it is reported they can enhance M1 macrophages modified to M2 macrophages in SCI. Caron et al. [138] applied the functional sequence RDG to modified agarose hydrogels and implanted them into a rat SCI model. Their results showed that the hydrogels can not only repair injured spinal cord but also be able to increase and/or convert efficaciously M2 macrophages in the injured site, promoting a proregenerative environment that represents a relevant outcome in treating SCI. Chedly et al. [139] also found that chitosan favors tissue repair in part by increasing activation and/or proliferation of M2 macrophages during the early postlesion phase. Recently, experimental evidence has demonstrated that the imidazole-poly hydrogel promotes ECM remodeling by activating the metalloproteinase-9 (MMP-9) matrix found in macrophages. This indicates that hydrogels may perform complex interactions with the immune system during SCI treatment [140]. However, the mechanisms of increased proliferation of M2 macrophages after applying hydrogels are still not elucidated.

In summary, hydrogels have great potential in the treatment of SCI. They have advantages such as excellent histological and functional recovery and the fact that they can be injected into lesions. The injectability of hydrogels minimizes the risk of secondary injury when hydrogels are administrated in SCI. They can also be modified by functional sequences or delivering growth factors. However, issues such as the need to enhance their mechanical strength, durability, and stability in application and balance between fluidity and mechanical strength need to be investigated in future studies. The exact mechanisms by which hydrogels interact with SCI also require further study.

### 3.2. Application of Biodegradable Scaffolds in SCI

Biodegradable scaffolds are also important biomaterials that are utilized in SCI. They are often surgically implanted into lesions and are synthesized through electrospinning techniques to decrease the use of organic solvent. In the spinal cord, the axons often appear in a longitude arrangement, and the electrospinning technique can fabric materials into any desired pattern and mimic the arrangement of axons. Chitosan, gelatin, PCL, and PLGA are the scaffolds predominantly applied in SCI, as they have an effect on axon regeneration, are anti-inflammatory, and promote tissue repair [141]. The effects of gelatin and PLGA scaffolds have been compared by Du et al. Their results suggest that gelatin scaffolds are superior to PLGA scaffold in SCI treatment, possibly because PLGA scaffolds generate more acidic medium than gelatin scaffolds in the process of degradation [142].

Biodegradable scaffolds can be incorporated with hydrogels to treat SCI [24]. The goal of this approach is to combine the therapeutic ability of hydrogels with the mechanical and physical properties of biodegradable scaffolds to enhance treatment effects. Gelain et al. [143] developed PCL/PLGA nanostructured microguidance scaffolds synthesized through the electrospinning technique. They implanted the scaffolds into chronic rat SCI lesions with self-assembled RADA16-I-BMHP1. Their results indicate that scaffolds can induce both regeneration and myelination of axons in chronic SCI and the motor function can also be recovered. The biodegradable scaffolds can also carry drugs or growth factors. Furthermore, they can be designed hierarchically; growth factors or functional materials can be synthesized in different layers of the scaffold; thus, with degradation of the scaffolds, they can take effect in different phases in SCI treatment. Thomas and Shea [144] implanted electrospun poly(lactide-co-glycolide) (PLG) scaffolds to carry polysaccharides, chitosan, and heparin. They found that, in the early stage of SCI, the scaffold can have an anti-inflammatory effect, after which the scaffolds can enhance axon growth and myelination. Neurotrophins-3 are applied in SCI treatment as they can encourage axon regeneration and cell proliferation. Fan et al. [145] synthesized PLGA/recombinant human neurotrophin-3 (rhNT3) scaffolds and utilized them in a rat SCI model. Their results indicated that axonal regeneration, locomotor, and sensory recovery occurred.

Surface modification of scaffolds can enhance the effect of regeneration through promotion of cell adhesion to the scaffold. Zamani et al. [146] developed electrospun PGLA three-dimensional core-sheath scaffolds. The developed scaffolds have a nanorough sheath and an aligned core. They implanted the developed scaffolds into an experimental SCI model and the results showed that they can improve axon regeneration as well as locomotor and sensory recovery. The pattern of the scaffold is another important parameter that can affect regeneration. It has been suggested that fabricating scaffolds with smaller diameter channels promotes greater regeneration over larger diameter channels [147].

Biodegradable scaffolds are also utilized in SCI as they have good mechanical strength and tunable inner pattern and are biodegradable. However, the need for surgical implantation narrows their application in some clinical situations. In summary, biodegradable scaffolds as biomaterials that are applied in SCI have considerable potential. In future studies, the application of new materials, relationship of the inner pattern and SCI recovery, exploration of multicomponent scaffolds, and development of a mini-invasive implantation method may be the main problems explored in the development of biodegradable scaffolds.

## 4. Biomaterial Scaffolds in Brain Regeneration

Traumatic brain injury (TBI), brain tumors, and brain hemorrhages are common causes of brain damage. In the USA, at least 5.3 million people suffered from disability after TBI, costing approximately $76.5 billion in lost productivity in 2010 [4]. These disabilities result in social and economic burdens and need to be solved urgently. The brain is the most complex organ in the human body. It has numerous neuronal cells and their neurites are woven into a sophisticated net. After injury, activation of the immune system and a poor instinctive repair process make it difficult to regenerate injured tissue. Hence, current strategies for brain tissue regeneration are still insufficient. Recently, some studies have applied biomaterial scaffolds to brain injury. Their results indicate that biomaterial scaffolds have significant potential in the treatment of brain injury. In this section, we review biomaterial scaffolds that have been applied in the treatment of TBI and other brain injury models. Biomaterials scaffolds that are applied with cell therapy for brain repair are discussed in Section 5.

### 4.1. Application of Hydrogel in Brain Regeneration

The brain is protected by the cranial bone, which makes it difficult to inject materials into the brain directly. The materials applied in brain injury scenarios are often surgically implanted. Natural materials, such as hyaluronic acid (HA) [148], collagen [149], chitosan, and methylcellulose [150, 151], have been used to synthesize hydrogels that are applied in these cases. Hydrogels can fill the brain cavity, replacing the growth-prohibiting environment with a more growth-permissive one. Further, it has been reported that hydrogels can decrease inflammation through reduction of secretion of inflammatory cytokine [152]. These mechanisms might enable cells and axons to infiltrate into hydrogels and further repair injured brain tissue [148]. Similar to the hydrogels applied in SCI, the hydrogels used in the brain can also be connected with functional peptides such as IKVAV and RGD to enhance their cell adhesion and axon regrowth effects [123]. In addition, hydrogels can be modified to carry antibody or drugs to improve regeneration. The Nogo-66 antibody carried HA hydrogels to promote axon regeneration in the rats stroke model; it has also been proven that these hydrogels have the effect of functional recovery [153]. Ma et al. synthesized HA based biodegradable hydrogel scaffolds and mixed them with PLGA microspheres containing vascular endothelial growth factor (VEGF), angiopoietin-1 (Ang1), and Nogo receptor antibody (NgR-Ab). They implanted the hydrogels into a mice brain ischemic model, and their results showed that the hydrogels have good compatibility with brain tissue and inhibition to gliosis and inflammation after implantation [154]. Recently, thermosensitive and sound-sensitive hydrogels have been developed for injection in brain injury. Koivisto et al. developed biomimetic hydrogels based on gellan gum. The developed hydrogels use bioamines spermidine and spermine to function as crosslinkers for gellan gum hydrogel at +37°C [155]. These hydrogels can promote neuronal cell migration, maturation, and neurite formation. Fernández-García et al. developed in situ gelling silk fibroin hydrogels. The gelation of silk fibroin solutions can be induced by sonication. These hydrogels can be injected into a mouse brain and integration of hydrogels into the brain tissue can be controlled by the intensity and duration of sonication. Their results prove that hydrogels have good biocompatibility in the brain and can be further applied in TBI treatment [156].

Several hydrogels made from synthetic materials have also been applied in brain regeneration. In general, such hydrogels are combined with cell therapy in brain injury treatment. Hydrogels made from synthetic materials are easier to chemically modify and have a 3D inner structure and low immune responses. PHPMA-RGD hydrogels containing brain-derived neurotrophic factors have been tested in a rat TBI model, with results showing the occurrence of axon regeneration and cell infiltration [157]. Self-assembling hydrogels, such as the RADA16-I hydrogel, also show the ability to promote regeneration of brain tissue and angiogenesis [158, 159]. Hydrogels made from synthetic materials can be combined with the scaffold to increase its strength. Polymer poly-L-lactide (PLLA) electrospun fibers with fibronectin inclusion and which are dispersed in an agarose/methylcellulose hydrogen can promote cell infiltration into the lesion site following brain injury [151].

### 4.2. Application of Biodegradable Scaffolds in Brain Regeneration

Biodegradable scaffolds are used to carry cells in brain regeneration. Only a few studies have investigated the effect of bare biodegradable scaffolds in animal TBI models. In this section, we discuss research conducted on the materials associated with brain regeneration.

The mechanisms of biodegradable scaffolds in promoting brain regeneration are mainly concentrated on their effects in enhancing support for microenvironments, guiding axon sprouting, and cell migration. PCL based scaffolds are the most studied scaffolds in brain regeneration. Nisbet et al. implanted electrospun PCL scaffolds into the caudate putamen of an adult rat brain and discovered neurite infiltration and growth in the scaffold [160]. They stated that the characteristics of the inner structure of PCL scaffolds, such as large porosity and perpendicular alignment at the implant-tissue interface, can promote neurite growth [161]. Wong et al. further studied the relationship between PCL scaffolds' channel direction and cell infiltration. Their study revealed that pores or channels oriented towards the parenchyma will increase astrocytic infiltration and that microgrooves oriented in the desired direction of cell migration and neuronal alignment will also provide benefit for regeneration. They also discovered that fully interconnecting channels for cell migration and tissue integration can increase regeneration [162]. Wong et al. also compared the regeneration effects of PCL and PLGA scaffolds in a rat brain. They found that both polymers can alleviate astrocytic activation, prevent enlargement of the defect, and improve neural ingrowth. However, PCL induces a lower inflammatory response than PLGA [163]. Recently, studies have indicated that migration and differentiation of endogenous stem cells play an important role in brain repair. Fon et al. applied electrospun PCL scaffolds incorporated with small molecule nonpeptide ligand (BDNF-mimetic) to a rat model. Their results proved that PCL scaffolds can improve neuroblast survival and promote neuroblast migration towards lesions [164]. Our team also investigated the effect of waterborne biodegradable polyurethane (WBPU) 3D porous scaffolds on the regeneration of a rat TBI model. We found that the scaffold can improve axonal regeneration as well as functional recovery. We also found that a percentage of poly ethylene glycol (PEG) within the scaffold may affect the result of regeneration [165]. The mechanisms underlying these phenomena are still being studied.

## 5. Combination of Biomaterial Scaffolds and Cell Therapy

The combination of biomaterial scaffolds and cell therapy in CNS regeneration has garnered the attention of researchers in recent years. The combination of these two therapeutic methods makes it possible to achieve both cell regeneration and tissue reconstruction. The basic principle of this modality is combining exogenous cell and scaffolds to form “live” scaffolds. These “live” scaffolds can be implanted into animals through injection or surgical implantation. The parenchyma part of CNS comprises neuron and glial cells that include astrocyte and oligodendrocyte. Neural stem/progenitor cells (NSPCs) are present in the adult CNS and are important in the maintenance and repair of CNS [226]. NSPCs can be differentiated into neuron and glial cells and hold great promise for repair of CNS [227]. However, NSPCs also have defects such as poor survival and uncontrolled differentiation. NSPCs have even been implicated as the origin of brain tumors [228, 229]. Thus, the survival factors and niches of NSPCs are critical for their application [230]. Biomaterial scaffolds have features that mimic the ECM and create a stable environment. Further, they have the potential to carry cytokines such as neural growth factor (NGF) or other functional molecules. Thus, biomaterial scaffolds are suitable for assisting with stem cell survival and differentiation [231]. In addition to NSPCs, other stem cells that have the potential to differentiate into neurocytes have also been implanted into biomaterial scaffold to help with CNS regeneration. These cells can be derived from bone marrow stem cells [138], induced pluripotent stem cells (iPSC) [232], induced pluripotent stem cells (iPSC) [233], embryonic stem cell [234], or adult stem cells [235]. The feasibility of transplantation of exogenous NSPCs has been tested by Li et al. who synthesized a methacrylamide chitosan (MAC) hydrogel system. They immobilized recombinant fusion proteins into methacrylamide chitosan (MAC) based biopolymer through a streptavidin linker. Their results indicated that the system can induce a majority of NSPCs to differentiate into the desired cell types by day 28. Their study proved that biomaterial scaffolds can regulate cells to differentiate into desired cells [236]. Biomaterial scaffolds can serve as carriers of NPSCs for injury treatment. They can create a stable microenvironment and provide the appropriate infrastructure to support cell migration into surrounding tissue [237]. In this section, we discuss progress made in the field of combination of biomaterial scaffolds and cell therapy in CNS regeneration.

In the field of SCI treatment, both hydrogels and biodegradable scaffolds have been studied in various studies. Hydrogels have been proven to improve both cell proliferation and differentiation* in vivo*. Further, they can carry growth factors or drugs to promote their effects in stem cell therapy. Mothe et al. developed a kind of hyaluronan and methyl cellulose (HAMC) hydrogels. They conjugated HAMC hydrogels with recombinant platelet-derived growth factor-A (rPDGF-A) to promote oligodendrocyte differentiation. The HAMC-rPDGF-A hydrogels were blended with adult brain-derived neural stem/progenitor cells (NSPCs), and the hydrogels were injected into a subacute, clinically relevant model of a rat SCI. They found that rats treated with HAMC-rPDGF-A hydrogels showed reduced lesion size, increased distribution of perilesional host neurons and oligodendrocytes, and better functional recovery [199]. An interesting comparison between the effects of hydrogels and biodegradable scaffolds in cell therapy has been made by Caron et al. They developed an agarose/carbomer based three-dimensional hydrogel and lyophilized sponge-like scaffolds, in which both scaffolds were loaded with mesenchymal stem cells (hMSC). Their results indicated that, compared with classic hydrogels, lyophilized sponge-like scaffolds can not only modulate inflammatory response, but also better preserve hMSC viability and stemness in an SCI mouse model [138]. This result indicates that biodegradable scaffolds may be better scaffolds in cell therapy. However, the controversy that stem cells can cause brain tumor is a long standing issue in cell therapy. Considering this problem, Führmann et al. developed a platelet-derived growth factor (PDGF-A) and RGD peptide modified hyaluronan and methylcellulose hydrogels. Their results showed that the hydrogels can enhance the survival of oligodendrocyte derived from iPSC. Moreover, they discovered that stem cells seeded in hydrogels attenuated the formation of teratoma, with the majority of stem cells differentiating to a glial phenotype. Their study indicates that hydrogels may decrease the formation of tumor after transplanting of stem cells, which is a profound result in stem cell therapy. However, more types and structures of materials need to be studied to confirm the phenomenon [238].

Biodegradable scaffolds have advantages in terms of mechanical property and biodegradability. Research on the application of biodegradable scaffolds in the treatment of SCI is concentrated on critical issues such as vitality of imbedded cells and whether they can differentiate into desired cell types. Terraf et al. utilized PCL scaffolds to carry human endometrial stem cells and applied them in a rat hemisected SCI model. According to their result, neurite outgrowth and axon regeneration can be observed and animals also showed functional recovery [239]. The strategy of combining different scaffolds to combine the advantage of each scaffold has also been used in cell therapy. Liu et al. implanted three-dimensional (3D) electrospun poly(lactide-co-glycolide)/polyethylene glycol (PLGA-PEG) scaffolds carrying iNSC into transected rat spinal cords. Their result showed iNSC survival and differentiation within the scaffolds. The cavity of the spinal cord was restored by the scaffold and functional recovery was also observed [217]. Kim et al. studied the difference in efficacy between implanted MSCs through traditional intralesional injection and through scaffold assisted implantation in a rat SCI model. They concentrated on engraftment and differentiation of transplanted cells, expression of neurotrophic factors in lesions, and functional recovery. Their results indicated higher success rate of MSCs engraftment in scaffold groups compared with the injection group. They also indicated that expression of neurotrophic factors is no different among all groups, whereas better functional recovery was exhibited in the scaffold groups. Their result proves the superiority of combining scaffolds and stem cells over traditional stem cell therapy. These results also imply that carrying neurotrophic factors in scaffolds seeded with stem cells may achieve better regeneration effects [240]. Neural growth factors (NGFs) are carried in biodegradable scaffolds that are supplied with stem cells to promote cell differentiation and proliferation. Among all NGFs, neurotrophin-3 (NT-3) is the most frequently used NGF in stem cell therapy. Johnson et al. reported that the combination of NT-3 and fibrin scaffolds can increase the total number of embryonic stem cell-derived neural progenitor cell (ESNPCs) derived neurons in NT-3 fibrin scaffolds after transplantation in a rat SCI model [241, 242]. Qiu et al. and Yang et al. both applied NT-3/chitosan scaffolds to promote the survival and proliferation of neural stem cells (NSCs). They exhibited that scaffolds can induce NSCs to differentiate into desired phenotypes such as neurons and astrocyte [243, 244]. Duan et al. further investigated the molecular mechanism underlying the phenomenon. Through weighted gene coexpression network analysis (WGCNA), they found that enhanced new neurogenesis and angiogenesis and reduced inflammatory responses were the key mechanisms of NT3-chitosan scaffolds in treating SCI [175].

Application of biomaterial scaffolds as cell carriers and tissue supporters has also been investigated in brain injury. Hydrogel use in the brain has been proved to promote proliferation, maturation, and differentiation of stem cells with or without other trophic factors. Because hydrogels are injectable, when damage is located in the deep region of the brain, they can be injected directly into lesions to avoid damage to the superficial cortical tissue. Shi et al. developed RADA16 self-assembling peptide hydrogels that carry brain-derived neurotrophic factor (BDNF). They seeded both MSCs and astrocytes into the scaffold and applied chemokine receptor 4 to promote migration of transplanted cells. Their results indicate that transplantation of scaffolds can aid repair of moderate-sized lesion cavities caused by TBI [245]. With the development of hydrogels, visualized stem cell hydrogels have been applied in the brain to monitor their in vivo process. Moshayedi et al. developed HA based self-polymerizing hydrogels that can be tracked* in vivo* through MRI imaging. They encapsulated human neural progenitor cells (iPS-NPCs) into the hydrogels and injected the hydrogels into a mice stroke model. Their results showed that hydrogels can promote survival of iPS-NPCs after transplantation into the stroke core. In addition, the hydrogels can also increase differentiation of transplanted cells [201]. Self-assembly hydrogels modified with functional peptides such as RADA16-IKVAV also have been reported to promote proliferation and differentiation of NSCs* in vivo* [246]. With the exception of SAPs, other self-assembly hydrogels such as thermosensitive diblock copolypeptide hydrogels (DCH) have also been applied to deliver NSCs. This shows that DCH can significantly increase the survival of NSCs in healthy CNS. In mouse models, DCT has also been distributed well in nonneural lesion cores, integrated with healthy neural cells at lesion perimeters, and supported the regrowing of host nerve fibers [247].

The application of biodegradable scaffolds and cell therapy in regeneration of the brain is a newly developed field and has been increasingly noticed in recent years. Chitosan scaffolds are one of the most popular scaffolds used in brain injury. Shi et al. developed a kind of BDNF blended chitosan scaffold to carry umbilical cord mesenchymal stem cells (hUC-MSCs) through a freeze-dry technique. They found that the scaffolds can increase the differentiation rate of NSCs and the average neuron perimeter [248]. The* in vivo* process of implanted cells in the brain is important for explaining the mechanisms of repair. To achieve this goal, Hwang et al. applied poly-L-lactic acid (PLLA) scaffolds to carry NSCs that express firefly luciferase. Thus, they can monitor the process of cell proliferation* in vivo* conveniently and noninvasively. Their result showed that the signals from cells in the scaffold are both stronger and more durable than nonencapsulated cells [249]. The plasma surface between scaffold and cells can affect cell adhesion and proliferation. Zandén et al. studied the effect of different plasma surfaces of polyurethane scaffolds for attachment and proliferation of human embryonic stem cell (hESC). They found that, compared with oxygen and hydrogen plasma surface, argon plasma induced the most optimal combination of surface functionality and roughness for cell expansion [250].

In summary, in the treatment of CNS damage, using both hydrogels and biodegradable stem cell scaffolds can combine advantages of both modalities. The scaffolds can increase the survival rate of stem cells and accelerate the accumulation of ECM. They also give stem cells an isolated environment to differentiate and proliferate. Moreover, stem cells can differentiate into desired cell types to reconstruct the damaged tissue and result in functional recovery. However, many factors can affect the repair effect, such as cell type, topography, category of materials, and physical and chemical properties of materials. Thus, the optimum method of combination of materials and stem cells still needs future study.

## 6. Conclusion and Prospects

In this review, we summarized present development in the application of biomaterial scaffolds in central nervous system regeneration. We showed that some materials have great potential in CNS regeneration as well as the combination of materials and cell therapy in this field. Biomaterial scaffolds can reduce inflammation at injury sites and can also change the microenvironment of lesions. In addition, they can carry drugs and neurotrophic factors to enhance the effect of therapy. Moreover, combining biomaterial scaffolds and cell therapy can promote survival and differentiation of stem cells and reduce the side effect of cell therapy. Hence, biomaterial scaffolds-assisted therapy is a promising strategy in CNS regeneration. However, these effects of scaffolds are based on animal experimentation; human CNS injury is more complex and is still a great problem that needs to be solved by the overall medical world. Developing biomaterial scaffolds that are biodegradable, biocompatible, and mechanically flexible is still an important issue in CNS regeneration. Moreover, using hybrid knowledge of cell therapy, pharmaceutical therapy, and clinic technique to enhance the ability of biomaterial scaffolds in CNS regeneration is an important strategy to improve biomaterial scaffolds. Finally, degrading the speed of biomaterial scaffolds should correspond to differentiating the phase of tissue regeneration, so that they can be designed to have different functions in different stages of regeneration. With the development of materials and biology, it is reasonable to surmise that we can achieve perfect CNS regeneration in the near future.

## Figures and Tables

**Table 1:** Natural materials scaffold applied in CNS.

Material	Description	Application in SCI	Application in brain injury
Agarose	Hydrogel	Functional recovery, tissue repair, delivering neurotrophic factor, stem cell therapy [166]	
	Biodegradable scaffold	Functional recovery, tissue repair, delivering neurotrophic factor, axonal regeneration [167]	

Alginate	Hydrogel	Functional recovery, tissue repair, delivering neurotrophic factor [69, 168]	Axonal regeneration [169]
	Biodegradable scaffold	Functional recovery, tissue repair, stem cell therapy [170]	

Cellulose	Hydrogel	Function recovery, axonal regeneration, delivering neurotrophic factor.	Tissue repair, stem cell therapy, anti-inflammation [171]

Chitosan	Hydrogel	Function recovery, axonal regeneration, anti-inflammation, stem cell therapy [172]	Function recovery, axonal regeneration, delivering neurotrophic factor and drug [173, 174]
	Biodegradable scaffold	Function recovery, axonal regeneration, anti-inflammation, delivering neurotrophic factor, stem cell therapy [175, 176]	Tissue repair, anti-inflammation, stem cell therapy [177]

Collagen	Hydrogel	Axonal regeneration, tissue repair, delivering neurotrophic factor, stem cell therapy [178]	Cell survival, axonal regeneration, stem cell therapy [179]
	Biodegradable scaffold	Function recovery, axonal regeneration, tissue repair, stem cell therapy [180–182]	Function recovery, tissue repair, stem cell therapy [183–185]

Fibrin	Hydrogel	Cell survival, axonal regeneration [186, 187]	Function recovery, cell survival, anti-inflammation, stem cell therapy [156, 188]
	Biodegradable scaffold	Cell survival and proliferation, tissue repair, anti-inflammation, stem cell therapy [189, 190]	Tissue repair, stem cell therapy [191].

Gelatin	Hydrogel	Cell survival, function recovery, axonal regeneration, tissue repair [192]	Cell survival and proliferation, stem cell therapy [193, 194]
	Biodegradable scaffold	Functional recovery, tissue repair, delivering neurotrophic factor, stem cell therapy [195, 196]	Tissue repair, anti-inflammation, stem cell therapy [197, 198]

Hyaluronic acid	Hydrogel	Function recovery, axonal regeneration, tissue repair, anti-inflammation, delivering neurotrophic factor, stem cell therapy [62, 199, 200]	Cell survival, axonal regeneration, stem cell therapy [201]

Xyloglucan	Hydrogel	Axonal regeneration, tissue repair, stem cell therapy [202]	Axonal regeneration, tissue repair, stem cell survival [203]

**Table 2:** Synthetic materials scaffold applied in CNS.

Material	Description	Application in SCI	Application in brain injury
FGLmx	Hydrogel	Function recovery, axonal regeneration, stem cell therapy [204]	

Poly-*ε*-caprolactone	Hydrogel	Cell survival, delivering neurotrophic factor [205]	
	Biodegradable scaffold	Cell survival, stem cell therapy, functional recovery [206, 207]	Axonal regeneration, cell survival, functional recovery, stem cell therapy [161, 164]

Poly(ethylene glycol)	Hydrogel	Axonal regeneration, functional improvements, anti-inflammation, cell survival [208, 209]	Axonal regeneration, anti-inflammation, cell survival, delivering neurotrophic factor [210, 211]
	Biodegradable scaffold	Function recovery, axonal regeneration, anti-inflammation [212]	

Poly(hydroxyethyl methacrylate)	Hydrogel	Nerve tissue regeneration and functional recovery, stem cell therapy [121, 213]	Cell survival, axonal regeneration [214]

Poly(hydroxypropyl methacrylate)	Hydrogel	Function recovery, axonal regeneration, anti-inflammation, delivering neurotrophic factor, stem cell therapy [76, 215]	Axonal regeneration, anti-inflammation [216]

Poly(lactide-co-glycolic acid)	Biodegradable scaffold	Axonal regeneration, tissue repair, delivering neurotrophic factor, stem cell therapy [145, 217–219]	Axonal regeneration, tissue repair [220]

Polyurethane	Hydrogel	Cell survival, axonal regeneration, functional recovery, stem cell therapy [221]	Cell survival, axonal regeneration, functional recovery, stem cell therapy [221]
	Biodegradable scaffold		Cell survival, axonal regeneration, functional recovery, stem cell therapy [221]

Hydroxy ethyl methacrylate	Hydrogel	Stem cell therapy and axons repair [222]	

PuraMatrix	Hydrogel	Functional recovery, spinal repair, and neuronal regeneration [223, 224]	Stem cell therapy [225]

Imidazole-poly(organophosphazenes)	Hydrogel	Function recovery, axonal regeneration, anti-inflammation [140]	

## References

[1] Spahn D. R., Bouillon B., Cerny V. (2013). Management of bleeding and coagulopathy following major trauma: an updated European guideline. *Critical Care*.

[2] Naidech A. M., Bendok B. R., Tamul P. (2009). Medical complications drive length of stay after brain hemorrhage: a cohort study. *Neurocritical Care*.

[3] Geisler F. H., Coleman W. P., Benzel E., Ducker T., John Hurlbert R. (2002). Spinal cord injury. *The Lancet*.

[4] Alali A. S., Burton K., Fowler R. A. (2015). Economic evaluations in the diagnosis and management of traumatic brain injury: a systematic review and analysis of quality. *Value in Health*.

[5] Fu T. S. (2016). Health & economic burden of traumatic brain injury in the emergency department. *Canadian Journal of Neurological Sciences*.

[6] Orive G., Anitua E., Pedraz J. L., Emerich D. F. (2009). Biomaterials for promoting brain protection, repair and regeneration. *Nature Reviews Neuroscience*.

[7] Gyoneva S., Ransohoff R. M. (2015). Inflammatory reaction after traumatic brain injury: therapeutic potential of targeting cell-cell communication by chemokines. *Trends in Pharmacological Sciences*.

[8] Whitney N. P., Eidem T. M., Peng H., Huang Y., Zheng J. C. (2009). Inflammation mediates varying effects in neurogenesis: relevance to the pathogenesis of brain injury and neurodegenerative disorders. *Journal of Neurochemistry*.

[9] Ridet J. L., Malhotra S. K., Privat A., Gage F. H. (1997). Reactive astrocytes: cellular and molecular cues to biological function. *Trends in Neurosciences*.

[10] Scheller A., Bai X., Kirchhoff F. (2017). The role of the oligodendrocyte lineage in acute brain trauma. *Neurochemical Research*.

[11] Das S., Basu A. (2008). Inflammation: a new candidate in modulating adult neurogenesis. *Journal of Neuroscience Research*.

[12] Hart T., Brenner L., Clark A. N. (2011). Major and minor depression after traumatic brain injury. *Archives of Physical Medicine and Rehabilitation*.

[13] Lowenstein D. H. (2009). Epilepsy after head injury: an overview. *Epilepsia*.

[14] Chang E. H., Adorjan I., Mundim M. V., Sun B., Dizon M. L. V., Szele F. G. (2016). Traumatic brain injury activation of the adult subventricular zone neurogenic niche. *Frontiers in Neuroscience*.

[15] Ekdahl C. T., Kokaia Z., Lindvall O. (2009). Brain inflammation and adult neurogenesis: the dual role of microglia. *Neuroscience*.

[16] Lau L. W., Cua R., Keough M. B., Haylock-Jacobs S., Yong V. W. (2013). Pathophysiology of the brain extracellular matrix: a new target for remyelination. *Nature Reviews Neuroscience*.

[17] Zhu Y., Soderblom C., Trojanowsky M., Lee D.-H., Lee J. K. (2015). Fibronectin matrix assembly after spinal cord injury. *Journal of Neurotrauma*.

[18] Povlishock J. T., Katz D. I. (2005). Update of neuropathology and neurological recovery after traumatic brain injury. *The Journal of Head Trauma Rehabilitation*.

[19] Gaudet A. D., Popovich P. G. (2014). Extracellular matrix regulation of inflammation in the healthy and injured spinal cord. *Experimental Neurology*.

[20] Dixon K. J., Theus M. H., Nelersa C. M. (2015). Endogenous neural stem/progenitor cells stabilize the cortical microenvironment after traumatic brain injury. *Journal of Neurotrauma*.

[21] Hemphill M. A., Dauth S., Yu C. J., Dabiri B. E., Parker K. K. (2015). Traumatic brain injury and the neuronal microenvironment: a potential role for neuropathological mechanotransduction. *Neuron*.

[22] Drago D., Cossetti C., Iraci N. (2013). The stem cell secretome and its role in brain repair. *Biochimie*.

[23] Muheremu A., Peng J., Ao Q. (2016). Stem cell based therapies for spinal cord injury. *Tissue & Cell*.

[24] Saracino G. A. A., Cigognini D., Silva D., Caprini A., Gelain F. (2013). Nanomaterials design and tests for neural tissue engineering. *Chemical Society Reviews*.

[25] Stenudd M., Sabelström H., Frisén J. (2015). Role of endogenous neural stem cells in spinal cord injury and repair. *JAMA Neurology*.

[26] Gennai S., Monsel A., Hao Q. (2015). Cell-Based therapy for traumatic brain injury. *British Journal of Anaesthesia*.

[27] Acosta S. A., Tajiri N., Shinozuka K. (2013). Long-term upregulation of inflammation and suppression of cell proliferation in the brain of adult rats exposed to traumatic brain injury using the controlled cortical impact model. *PLoS ONE*.

[28] Halberstadt C., Emerich D. (2007). *Cellular Transplants: From Lab to Clinic*.

[29] Wu Y., Lin W., Hao H., Li J., Luo F., Tan H. (2017). Nanofibrous scaffold from electrospinning biodegradable waterborne polyurethane/poly(vinyl alcohol) for tissue engineering application. *Journal of Biomaterials Science, Polymer Edition*.

[30] Stratton S., Shelke N. B., Hoshino K., Rudraiah S., Kumbar S. G. (2016). Bioactive polymeric scaffolds for tissue engineering. *Bioactive Materials*.

[31] Shie M.-Y., Chang W.-C., Wei L.-J. (2017). 3D printing of cytocompatible water-based light-cured polyurethane with hyaluronic acid for cartilage tissue engineering applications. *Materials*.

[32] Di Liddo R., Paganin P., Lora S. (2014). Poly-epsilon-caprolactone composite scaffolds for bone repair. *International Journal of Molecular Medicine*.

[33] Singh A., Peppas N. A. (2014). Hydrogels and scaffolds for immunomodulation. *Advanced Materials*.

[34] Rice J. J., Martino M. M., De Laporte L., Tortelli F., Briquez P. S., Hubbell J. A. (2013). Engineering the regenerative microenvironment with biomaterials. *Advanced Healthcare Materials*.

[35] Skop N. B., Calderon F., Cho C. H., Gandhi C. D., Levison S. W. (2014). Improvements in biomaterial matrices for neural precursor cell transplantation. *Molecular and Cellular Therapies*.

[36] Gilbert T. W., Sellaro T. L., Badylak S. F. (2006). Decellularization of tissues and organs. *Biomaterials*.

[37] Shrestha B., Coykendall K., Li Y., Moon A., Priyadarshani P., Yao L. (2014). Repair of injured spinal cord using biomaterial scaffolds and stem cells. *Stem Cell Research & Therapy*.

[38] Meng F., Modo M., Badylak S. F. (2014). Biologic scaffold for CNS repair. *Journal of Regenerative Medicine*.

[39] Tam R. Y., Fuehrmann T., Mitrousis N., Shoichet M. S. (2014). Regenerative therapies for central nervous system diseases: a biomaterials approach. *Neuropsychopharmacology*.

[40] David S., Aguayo A. J. (1981). Axonal elongation into peripheral nervous system 'bridges' after central nervous system injury in adult rats. *Science*.

[41] Vollath D. (2013). *Nanomaterials*.

[42] Gleiter H. (2000). Nanostructured materials: basic concepts and microstructure. *Acta Materialia*.

[43] Amsler C., Doser M., Antonelli M. (2008). Review of particle physics. *Physics Letters B*.

[44] Hoffman A. S. (2012). Hydrogels for biomedical applications. *Advanced Drug Delivery Reviews*.

[45] Fraczek-Szczypta A. (2014). Carbon nanomaterials for nerve tissue stimulation and regeneration. *Materials Science and Engineering C: Materials for Biological Applications*.

[46] Verma S., Domb A. J., Kumar N. (2011). Nanomaterials for regenerative medicine. *Nanomedicine*.

[47] Bradke F., Fawcett J. W., Spira M. E. (2012). Assembly of a new growth cone after axotomy: The precursor to axon regeneration. *Nature Reviews Neuroscience*.

[48] Gomis-Rüth S., Wierenga C. J., Bradke F. (2008). Plasticity of polarization: changing dendrites into axons in neurons integrated in neuronal circuits. *Current Biology*.

[49] Ellis-Behnke R. G., Teather L. A., Schneider G. E., So K.-F. (2007). Using nanotechnology to design potential therapies for CNS regeneration. *Current Pharmaceutical Design*.

[50] Acarregui A., Pedraz J. L., Blanco F. J., Hernández R. M., Orive G. (2013). Hydrogel-based scaffolds for enclosing encapsulated therapeutic cells. *Biomacromolecules*.

[51] Collins M. N., Birkinshaw C. (2013). Hyaluronic acid based scaffolds for tissue engineering—a review. *Carbohydrate Polymers*.

[52] Khaing Z. Z., Ehsanipour A., Hofstetter C. P., Seidlits S. K. (2016). Injectable hydrogels for spinal cord repair: a focus on swelling and intraspinal pressure. *Cells Tissues Organs*.

[53] Suzuki A., Sasaki S. (2015). Swelling and mechanical properties of physically crosslinked poly(vinyl alcohol) hydrogels. *Proceedings of the Institution of Mechanical Engineers, Part H: Journal of Engineering in Medicine*.

[54] Acarregui A., Herrán E., Igartua M. (2014). Multifunctional hydrogel-based scaffold for improving the functionality of encapsulated therapeutic cells and reducing inflammatory response. *Acta Biomaterialia*.

[55] Garg T., Goyal A. K. (2014). Biomaterial-based scaffolds—current status and future directions. *Expert Opinion on Drug Delivery*.

[56] Luo Y., Shoichet M. S. (2004). A photolabile hydrogel for guided three-dimensional cell growth and migration. *Nature Materials*.

[57] Patenaude M., Smeets N. M. B., Hoare T. (2014). Designing injectable, covalently cross-linked hydrogels for biomedical applications. *Macromolecular Rapid Communications*.

[58] Bakarich S. E., Pidcock G. C., Balding P., Stevens L., Calvert P., Panhuis M. I. H. (2012). Recovery from applied strain in interpenetrating polymer network hydrogels with ionic and covalent cross-links. *Soft Matter*.

[59] Kartha K. K., Babu S. S., Srinivasan S., Ajayaghosh A. (2012). Attogram sensing of trinitrotoluene with a self-assembled molecular gelator. *Journal of the American Chemical Society*.

[60] Hosseinkhani H., Hong P.-D., Yu D.-S. (2013). Self-assembled proteins and peptides for regenerative medicine. *Chemical Reviews*.

[61] Macaya D., Spector M. (2012). Injectable hydrogel materials for spinal cord regeneration: a review. *Biomedical Materials*.

[62] Führmann T., Obermeyer J., Tator C. H., Shoichet M. S. (2015). Click-crosslinked injectable hyaluronic acid hydrogel is safe and biocompatible in the intrathecal space for ultimate use in regenerative strategies of the injured spinal cord. *Methods*.

[63] Koh L.-D., Cheng Y., Teng C.-P. (2015). Structures, mechanical properties and applications of silk fibroin materials. *Progress in Polymer Science*.

[64] Fini M., Motta A., Torricelli P. (2005). The healing of confined critical size cancellous defects in the presence of silk fibroin hydrogel. *Biomaterials*.

[65] Liu Z., Wang H., Wang Y. (2012). The influence of chitosan hydrogel on stem cell engraftment, survival and homing in the ischemic myocardial microenvironment. *Biomaterials*.

[66] Alvarez G. S., Hélary C., Mebert A. M., Wang X., Coradin T., Desimone M. F. (2014). Antibiotic-loaded silica nanoparticle-collagen composite hydrogels with prolonged antimicrobial activity for wound infection prevention. *Journal of Materials Chemistry B*.

[67] Tabata Y., Miyao M., Ozeki M., Ikada Y. (2000). Controlled release of vascular endothelial growth factor by use of collagen hydrogels. *Journal of Biomaterials Science, Polymer Edition*.

[68] Desai R. M., Koshy S. T., Hilderbrand S. A., Mooney D. J., Joshi N. S. (2015). Versatile click alginate hydrogels crosslinked via tetrazine-norbornene chemistry. *Biomaterials*.

[69] Ansorena E., de Berdt P., Ucakar B. (2013). Injectable alginate hydrogel loaded with GDNF promotes functional recovery in a hemisection model of spinal cord injury. *International Journal of Pharmaceutics*.

[70] Thiele J., Ma Y., Bruekers S. M. C., Ma S., Huck W. T. S. (2014). 25th anniversary article: designer hydrogels for cell cultures: a materials selection guide. *Advanced Materials*.

[71] Seliktar D. (2012). Designing cell-compatible hydrogels for biomedical applications. *Science*.

[72] Shapiro J. M., Oyen M. L. (2013). Hydrogel composite materials for tissue engineering scaffolds. *The Journal of The Minerals, Metals & Materials Society*.

[73] Peppas N. A., Moynihan H. J., Lucht L. M. (1985). The structure of highly crosslinked poly(2‐hydroxyethyl methacrylate) hydrogels. *Journal of Biomedical Materials Research Part B: Applied Biomaterials*.

[74] Ahmad M. B., Huglin M. B. (1994). DSC studies on states of water in crosslinked poly(methyl methacrylate‐co‐n‐vinyl‐2‐pyrrolidone) hydrogels. *Polymer International*.

[75] Almany L., Seliktar D. (2005). Biosynthetic hydrogel scaffolds made from fibrinogen and polyethylene glycol for 3D cell cultures. *Biomaterials*.

[76] Pertici V., Amendola J., Laurin J. (2013). The use of poly(N-[2-hydroxypropyl]- methacrylamide) hydrogel to repair a T10 spinal cord hemisection in rat: a behavioural, electrophysiological and anatomical examination. *ASN Neuro*.

[77] Alfurhood J. A., Sun H., Bachler P. R., Sumerlin B. S. (2016). Hyperbranched poly(N-(2-hydroxypropyl) methacrylamide) via RAFT self-condensing vinyl polymerization. *Polymer Chemistry*.

[78] Hackelbusch S., Rossow T., Steinhilber D., Weitz D. A., Seiffert S. (2015). Hybrid microgels with thermo-tunable elasticity for controllable cell confinement. *Advanced Healthcare Materials*.

[79] Chassenieux C., Tsitsilianis C. (2016). Recent trends in pH/thermo-responsive self-assembling hydrogels: from polyions to peptide-based polymeric gelators. *Soft Matter*.

[80] Cigognini D., Silva D., Paloppi S., Gelain F. (2014). Evaluation of mechanical properties and therapeutic effect of injectable self-assembling hydrogels for spinal cord injury. *Journal of Biomedical Nanotechnology*.

[81] Zhang S., Lockshin C., Herbert A., Winter E., Rich A. (1992). Zuotin, a putative Z-DNA binding protein in Saccharomyces cerevisiae. *EMBO Journal*.

[82] Wen Y., Roudebush S. L., Buckholtz G. A. (2014). Coassembly of amphiphilic peptide EAK16-II with histidinylated analogues and implications for functionalization of *β*-sheet fibrils in vivo. *Biomaterials*.

[83] Yokoi H., Kinoshita T., Zhang S. (2005). Dynamic reassembly of peptide RADA16 nanofiber scaffold. *Proceedings of the National Acadamy of Sciences of the United States of America*.

[84] Yu Z., Cai Z., Chen Q. (2016). Engineering *β*-sheet peptide assemblies for biomedical applications. *Biomaterials Science*.

[85] Gelain F., Bottai D., Vescovi A., Zhang S. (2006). Designer self-assembling peptide nanofiber scaffolds for adult mouse neural stem cell 3-dimensional cultures. *PLoS ONE*.

[86] Yigit S., Sanyal R., Sanyal A. (2011). Fabrication and functionalization of hydrogels through 'click' chemistry. *Chemistry*.

[87] Cai L., Heilshorn S. C. (2014). Designing ECM-mimetic materials using protein engineering. *Acta Biomaterialia*.

[88] Sun Y., Li W., Wu X. (2016). Functional self-assembling peptide nanofiber hydrogels designed for nerve degeneration. *ACS Applied Materials & Interfaces*.

[89] Kassam H., Moreira E., Moyer T., Stupp S., Kibbe M. (2013). Prevention of neointimal hyperplasia with systemic injection of a targeted drug-eluting peptide amphiphile. *Journal of Surgical Research*.

[90] Cui H., Webber M. J., Stupp S. I. (2010). Self-assembly of peptide amphiphiles: from molecules to nanostructures to biomaterials. *Biopolymers*.

[91] Nikkola L., Seppälä J., Harlin A., Ndreu A., Ashammakhi N. (2006). Electrospun multifunctional diclofenac sodium releasing nanoscaffold. *Journal of Nanoscience and Nanotechnology*.

[92] Wu X., Liu Y., Li X. (2010). Preparation of aligned porous gelatin scaffolds by unidirectional freeze-drying method. *Acta Biomaterialia*.

[93] Tan Y., Richards D. J., Trusk T. C. (2014). 3D printing facilitated scaffold-free tissue unit fabrication. *Biofabrication*.

[94] Lao L. L., Peppas N. A., Boey F. Y. C., Venkatraman S. S. (2011). Modeling of drug release from bulk-degrading polymers. *International Journal of Pharmaceutics*.

[95] Bat E., Zhang Z., Feijen J., Grijpma D. W., Poot A. A. (2014). Biodegradable elastomers for biomedical applications and regenerative medicine. *Journal of Regenerative Medicine*.

[96] Bitar K. N., Zakhem E. (2014). Design strategies of biodegradable scaffolds for tissue regeneration. *Biomedical Engineering and Computational Biology*.

[97] Mikos A. G., Temenoff J. S. (2000). Formation of highly porous biodegradable scaffolds for tissue engineering. *Electronic Journal of Biotechnology*.

[98] Guan J., Stankus J. J., Wagner W. R. (2007). Biodegradable elastomeric scaffolds with basic fibroblast growth factor release. *Journal of Controlled Release*.

[99] Babensee J. E., McIntire L. V., Mikos A. G. (2000). Growth factor delivery for tissue engineering. *Pharmaceutical Research*.

[100] Phipps M. C., Clem W. C., Grunda J. M., Clines G. A., Bellis S. L. (2012). Increasing the pore sizes of bone-mimetic electrospun scaffolds comprised of polycaprolactone, collagen I and hydroxyapatite to enhance cell infiltration. *Biomaterials*.

[101] Paşcu E. I., Stokes J., McGuinness G. B. (2013). Electrospun composites of PHBV, silk fibroin and nano-hydroxyapatite for bone tissue engineering. *Materials Science and Engineering C: Materials for Biological Applications*.

[102] Croisier F., Jérôme C. (2013). Chitosan-based biomaterials for tissue engineering. *European Polymer Journal*.

[103] Farrugia B. L., Brown T. D., Upton Z., Hutmacher D. W., Dalton P. D., Dargaville T. R. (2013). Dermal fibroblast infiltration of poly(*ε*-caprolactone) scaffolds fabricated by melt electrospinning in a direct writing mode. *Biofabrication*.

[104] Izal I., Aranda P., Sanz-Ramos P. (2013). Culture of human bone marrow-derived mesenchymal stem cells on of poly(l-lactic acid) scaffolds: potential application for the tissue engineering of cartilage. *Knee Surgery, Sports Traumatology, Arthroscopy*.

[105] Grad S., Kupcsik L., Gorna K., Gogolewski S., Alini M. (2003). The use of biodegradable polyurethane scaffolds for cartilage tissue engineering: potential and limitations. *Biomaterials*.

[106] Kweon H., Yoo M. K., Park I. K. (2003). A novel degradable polycaprolactone networks for tissue engineering. *Biomaterials*.

[107] Bini T. B., Gao S., Tan T. C. (2004). Electrospun poly(L-lactide-co-glycolide) biodegradable polymer nanofibre tubes for peripheral nerve regeneration. *Nanotechnology*.

[108] Chew S. Y., Mi R., Hoke A., Leong K. W. (2007). Aligned protein-polymer composite fibers enhance nerve regeneration: a potential tissue-engineering platform. *Advanced Functional Materials*.

[109] McDonald J. W., Sadowsky C. (2002). Spinal-cord injury. *The Lancet*.

[110] Joosten E. A. J., Bär P. R., Gispen W. H. (1995). Collagen implants and cortico‐spinal axonal growth after mid‐thoracic spinal cord lesion in the adult rat. *Journal of Neuroscience Research*.

[111] Johnson P. J., Parker S. R., Sakiyama-Elbert S. E. (2010). Fibrin-based tissue engineering scaffolds enhance neural fiber sprouting and delay the accumulation of reactive astrocytes at the lesion in a subacute model of spinal cord injury. *Journal of Biomedical Materials Research Part A*.

[112] Cho Y., Shi R., Borgens R. B. (2010). Chitosan produces potent neuroprotection and physiological recovery following traumatic spinal cord injury. *Journal of Experimental Biology*.

[113] Furuya T., Hashimoto M., Koda M. (2013). Treatment with basic fibroblast growth factorincorporated gelatin hydrogel does not exacerbate mechanical allodynia after spinal cord contusion injury in rats. *The Journal of Spinal Cord Medicine*.

[114] Chvatal S. A., Kim Y.-T., Bratt-Leal A. M., Lee H., Bellamkonda R. V. (2008). Spatial distribution and acute anti-inflammatory effects of Methylprednisolone after sustained local delivery to the contused spinal cord. *Biomaterials*.

[115] Jain A., McKeon R. J., Brady-Kalnay S. M., Bellamkonda R. V. (2011). Sustained delivery of activated rho GTPases and BDNF promotes axon growth in CSPG-rich regions following spinal cord injury. *PLoS ONE*.

[116] McKay C. A., Pomrenke R. D., McLane J. S. (2014). An injectable, calcium responsive composite hydrogel for the treatment of acute spinal cord injury. *ACS Applied Materials & Interfaces*.

[117] Wen Y., Yu S., Wu Y. (2016). Spinal cord injury repair by implantation of structured hyaluronic acid scaffold with PLGA microspheres in the rat. *Cell and Tissue Research*.

[118] Hejcl A., Lesny P., Pradny M. (2008). Biocompatible hydrogels in spinal cord injury repair. *Physiological Research*.

[119] Namba R. M., Cole A. A., Bjugstad K. B., Mahoney M. J. (2009). Development of porous PEG hydrogels that enable efficient, uniform cell-seeding and permit early neural process extension. *Acta Biomaterialia*.

[120] Hejcl A., Urdzikova L., Sedy J. (2008). Acute and delayed implantation of positively charged 2-hydroxyethyl methacrylate scaffolds in spinal cord injury in the rat. *Journal of Neurosurgery: Spine*.

[121] Chen B., He J., Yang H. (2015). Repair of spinal cord injury by implantation of bFGF-incorporated HEMA-MOETACL hydrogel in rats. *Scientific Reports*.

[122] Cui F. Z., Tian W. M., Hou S. P., Xu Q. Y., Lee I.-S. (2006). Hyaluronic acid hydrogel immobilized with RGD peptides for brain tissue engineering. *Journal of Materials Science: Materials in Medicine*.

[123] Wei Y. T., Tian W. M., Yu X. (2007). Hyaluronic acid hydrogels with IKVAV peptides for tissue repair and axonal regeneration in an injured rat brain. *Biomedical Materials*.

[124] Hou S., Xu Q., Tian W. (2005). The repair of brain lesion by implantation of hyaluronic acid hydrogels modified with laminin. *Journal of Neuroscience Methods*.

[125] Woerly S., Pinet E., de Robertis L., van Diep D., Bousmina M. (2001). Spinal cord repair with PHPMA hydrogel containing RGD peptides (NeuroGel). *Biomaterials*.

[126] Guo J., Su H., Zeng Y. (2007). Reknitting the injured spinal cord by self-assembling peptide nanofiber scaffold. *Nanomedicine: Nanotechnology, Biology and Medicine*.

[127] Cigognini D., Satta A., Colleoni B. (2011). Evaluation of early and late effects into the acute spinal cord injury of an injectable functionalized self-assembling scaffold. *PLoS ONE*.

[128] Tysseling V. M., Sahni V., Pashuck E. T. (2010). Self-assembling peptide amphiphile promotes plasticity of serotonergic fibers following spinal cord injury. *Journal of Neuroscience Research*.

[129] Semple B. D., Frugier T., Morganti-Kossmann M. C. (2010). CCL2 modulates cytokine production in cultured mouse astrocytes. *Journal of Neuroinflammation*.

[130] Ogle M. E., Segar C. E., Sridhar S., Botchwey E. A. (2016). Monocytes and macrophages in tissue repair: implications for immunoregenerative biomaterial design. *Experimental Biology and Medicine*.

[131] Spiller K. L., Anfang R. R., Spiller K. J. (2014). The role of macrophage phenotype in vascularization of tissue engineering scaffolds. *Biomaterials*.

[132] Spiller K. L., Nassiri S., Witherel C. E. (2015). Sequential delivery of immunomodulatory cytokines to facilitate the M1-to-M2 transition of macrophages and enhance vascularization of bone scaffolds. *Biomaterials*.

[133] Gordon S., Taylor P. R. (2005). Monocyte and macrophage heterogeneity. *Nature Reviews Immunology*.

[134] Shi C., Pamer E. G. (2011). Monocyte recruitment during infection and inflammation. *Nature Reviews Immunology*.

[135] Gensel J. C., Zhang B. (2015). Macrophage activation and its role in repair and pathology after spinal cord injury. *Brain Research*.

[136] Kim C. C., Nakamura M. C., Hsieh C. L. (2016). Brain trauma elicits non-canonical macrophage activation states. *Journal of Neuroinflammation*.

[137] Makinde H. M., Cuda C. M., Just T. B., Perlman H. R., Schwulst S. J. (2017). Nonclassical monocytes mediate secondary injury, neurocognitive outcome, and neutrophil infiltration after traumatic brain injury. *The Journal of Immunology*.

[138] Caron I., Rossi F., Papa S. (2016). A new three dimensional biomimetic hydrogel to deliver factors secreted by human mesenchymal stem cells in spinal cord injury. *Biomaterials*.

[139] Chedly J., Soares S., Montembault A. (2017). Physical chitosan microhydrogels as scaffolds for spinal cord injury restoration and axon regeneration. *Biomaterials*.

[140] Hong L. T. A., Kim Y.-M., Park H. H. (2017). An injectable hydrogel enhances tissue repair after spinal cord injury by promoting extracellular matrix remodeling. *Nature Communications*.

[141] Sakiyama-Elbert S., Johnson P. J., Hodgetts S. I., Plant G. W., Harvey A. R. (2012). Scaffolds to promote spinal cord regeneration. *Handbook of Clinical Neurology*.

[142] Du B.-L., Zeng C.-G., Zhang W., Quan D.-P., Ling E.-A., Zeng Y.-S. (2014). A comparative study of gelatin sponge scaffolds and PLGA scaffolds transplanted to completely transected spinal cord of rat. *Journal of Biomedical Materials Research Part A*.

[143] Gelain F., Panseri S., Antonini S. (2011). Transplantation of nanostructured composite scaffolds results in the regeneration of chronically injured spinal cords. *ACS Nano*.

[144] Thomas A. M., Shea L. D. (2013). Polysaccharide-modified scaffolds for controlled lentivirus delivery in vitro and after spinal cord injury. *Journal of Controlled Release*.

[145] Fan J., Zhang H., He J. (2011). Neural regrowth induced by PLGA nerve conduits and neurotrophin-3 in rats with complete spinal cord transection. *Journal of Biomedical Materials Research Part B: Applied Biomaterials*.

[146] Zamani F., Amani-Tehran M., Latifi M., Shokrgozar M. A., Zaminy A. (2014). Promotion of spinal cord axon regeneration by 3D nanofibrous core-sheath scaffolds. *Journal of Biomedical Materials Research Part A*.

[147] Krych A. J., Rooney G. E., Chen B. (2009). Relationship between scaffold channel diameter and number of regenerating axons in the transected rat spinal cord. *Acta Biomaterialia*.

[148] Cook D. J., Nguyen C., Chun H. N. (2016). Hydrogel-delivered brain-derived neurotrophic factor promotes tissue repair and recovery after stroke. *Journal of Cerebral Blood Flow & Metabolism*.

[149] Guan J., Zhu Z., Zhao R. C. (2013). Transplantation of human mesenchymal stem cells loaded on collagen scaffolds for the treatment of traumatic brain injury in rats. *Biomaterials*.

[150] Richter A., Xie Y., Schumacher A. (2013). A simple implantation method for flexible, multisite microelectrodes into rat brains. *Frontiers in Neuroengineering*.

[151] Rivet C. J., Zhou K., Gilbert R. J., Finkelstein D. I., Forsythe J. S. (2015). Cell infiltration into a 3D electrospun fiber and hydrogel hybrid scaffold implanted in the brain. *Biomatter*.

[152] Sirova M., Vlierberghe S. V., Matyasova V. (2014). Immunocompatibility evaluation of hydrogel-coated polyimide implants for applications in regenerative medicine. *Journal of Biomedical Materials Research Part A*.

[153] Ma J., Tian W.-M., Hou S.-P., Xu Q.-Y., Spector M., Cui F.-Z. (2007). An experimental test of stroke recovery by implanting a hyaluronic acid hydrogel carrying a Nogo receptor antibody in a rat model. *Biomedical Materials*.

[154] Ju R., Wen Y., Gou R., Wang Y., Xu Q. (2014). The experimental therapy on brain ischemia by improvement of local angiogenesis with tissue engineering in the mouse. *Cell Transplantation*.

[155] Koivisto J. T., Joki T., Parraga J. E. (2017). Bioamine-crosslinked gellan gum hydrogel for neural tissue engineering. *Biomedical Materials*.

[156] Fernández-García L., Marí-Buyé N., Barios J. A. (2016). Safety and tolerability of silk fibroin hydrogels implanted into the mouse brain. *Acta Biomaterialia*.

[157] Loh N. K., Woerly S., Bunt S. M., Wilton S. D., Harvey A. R. (2001). The regrowth of axons within tissue defects in the cns is promoted by implanted hydrogel matrices that contain BDNF and CNTF producing fibroblasts. *Experimental Neurology*.

[158] Park K. I., Teng Y. D., Snyder E. Y. (2002). The injured brain interacts reciprocally with neural stem cells supported by scaffolds to reconstitute lost tissue. *Nature Biotechnology*.

[159] Wang T., Chang K., Chen L., Liao S., Yeh C., Chuang Y. (2017). Effects of an injectable functionalized self-assembling nanopeptide hydrogel on angiogenesis and neurogenesis for regeneration of the central nervous system. *Nanoscale*.

[160] Nisbet D. R., Yu L. M. Y., Zahir T., Forsythe J. S., Shoichet M. S. (2008). Characterization of neural stem cells on electrospun poly(*ε*- caprolactone) submicron scaffolds: evaluating their potential in neural tissue engineering. *Journal of Biomaterials Science, Polymer Edition*.

[161] Nisbet D. R., Rodda A. E., Horne M. K., Forsythe J. S., Finkelstein D. I. (2009). Neurite infiltration and cellular response to electrospun polycaprolactone scaffolds implanted into the brain. *Biomaterials*.

[162] Wong D. Y., Krebsbach P. H., Hollister S. J. (2008). Brain cortex regeneration affected by scaffold architectures: laboratory investigation. *Journal of Neurosurgery*.

[163] Wong D. Y., Hollister S. J., Krebsbach P. H., Nosrat C. (2007). Poly(*ε*-caprolactone) and poly (L-lactic-co-glycolic acid) degradable polymer sponges attenuate astrocyte response and lesion growth in acute traumatic brain injury. *Tissue Engineering Part A*.

[164] Fon D., Zhou K., Ercole F. (2014). Nanofibrous scaffolds releasing a small molecule BDNF-mimetic for the re-direction of endogenous neuroblast migration in the brain. *Biomaterials*.

[165] Wang Y.-C., Fang F., Wu Y.-K. (2016). Waterborne biodegradable polyurethane 3-dimensional porous scaffold for rat cerebral tissue regeneration. *RSC Advances*.

[166] Jain A., Kim Y.-T., McKeon R. J., Bellamkonda R. V. (2006). In situ gelling hydrogels for conformal repair of spinal cord defects, and local delivery of BDNF after spinal cord injury. *Biomaterials*.

[167] Gao M., Lu P., Bednark B. (2013). Templated agarose scaffolds for the support of motor axon regeneration into sites of complete spinal cord transection. *Biomaterials*.

[168] Des Rieux A., De Berdt P., Ansorena E. (2014). Vascular endothelial growth factor-loaded injectable hydrogel enhances plasticity in the injured spinal cord. *Journal of Biomedical Materials Research Part A*.

[169] Pawar K., Prang P., Müller R. (2015). Intrinsic and extrinsic determinants of central nervous system axon outgrowth into alginate-based anisotropic hydrogels. *Acta Biomaterialia*.

[170] Hosseini S. M., Sharafkhah A., Koohi-Hosseinabadi O., Semsar-Kazerooni M. (2016). Transplantation of neural stem cells cultured in alginate scaffold for spinal cord injury in rats. *Asian Spine Journal*.

[171] Wang Y., Cooke M. J., Morshead C. M., Shoichet M. S. (2012). Hydrogel delivery of erythropoietin to the brain for endogenous stem cell stimulation after stroke injury. *Biomaterials*.

[172] Nawrotek K., Marqueste T., Modrzejewska Z., Zarzycki R., Rusak A., Decherchi P. (2017). Thermogelling chitosan lactate hydrogel improves functional recovery after a C2 spinal cord hemisection in rat. *Journal of Biomedical Materials Research Part A*.

[173] Azadi A., Hamidi M., Rouini M.-R. (2013). Methotrexate-loaded chitosan nanogels as 'Trojan Horses' for drug delivery to brain: Preparation and in vitro/in vivo characterization. *International Journal of Biological Macromolecules*.

[174] Mo L., Yang Z., Zhang A., Li X. (2010). The repair of the injured adult rat hippocampus with NT-3-chitosan carriers. *Biomaterials*.

[175] Duan H., Ge W., Zhang A. (2015). Transcriptome analyses reveal molecular mechanisms underlying functional recovery after spinal cord injury. *Proceedings of the National Academy of Sciences of the USA*.

[176] Zhang J., Lu X., Feng G. (2016). Chitosan scaffolds induce human dental pulp stem cells to neural differentiation: potential roles for spinal cord injury therapy. *Cell and Tissue Research*.

[177] Huang C., Zhao L., Gu J. (2016). The migration and differentiation of hUC-MSCsCXCR4/GFP encapsulated in BDNF/chitosan scaffolds for brain tissue engineering. *Biomedical Materials*.

[178] Macaya D. J., Hayakawa K., Arai K., Spector M. (2013). Astrocyte infiltration into injectable collagen-based hydrogels containing FGF-2 to treat spinal cord injury. *Biomaterials*.

[179] Gil V., Del Río J. A. (2012). Analysis of axonal growth and cell migration in 3D hydrogel cultures of embryonic mouse CNS tissue. *Nature Protocols*.

[180] Han S., Wang B., Jin W. (2014). The collagen scaffold with collagen binding BDNF enhances functional recovery by facilitating peripheral nerve infiltrating and ingrowth in canine complete spinal cord transection. *Spinal Cord*.

[181] Altinova H., Möllers S., Führmann T. (2014). Functional improvement following implantation of a microstructured, type-I collagen scaffold into experimental injuries of the adult rat spinal cord. *Brain Research*.

[182] Li X., Xiao Z., Han J. (2013). Promotion of neuronal differentiation of neural progenitor cells by using EGFR antibody functionalized collagen scaffolds for spinal cord injury repair. *Biomaterials*.

[183] Huang K.-F., Hsu W.-C., Chiu W.-T., Wang J.-Y. (2012). Functional improvement and neurogenesis after collagen-GAG matrix implantation into surgical brain trauma. *Biomaterials*.

[184] Duan H., Li X., Wang C. (2016). Functional hyaluronate collagen scaffolds induce NSCs differentiation into functional neurons in repairing the traumatic brain injury. *Acta Biomaterialia*.

[185] Elias P. Z., Spector M. (2012). Implantation of a collagen scaffold seeded with adult rat hippocampal progenitors in a rat model of penetrating brain injury. *Journal of Neuroscience Methods*.

[186] Bayat N., Ebrahimi-Barough S., Ardakan M. M. M. (2016). Erratum to: differentiation of human endometrial stem cells into schwann cells in fibrin hydrogel as 3D culture. *Molecular Neurobiology*.

[187] Soleimannejad M., Ebrahimi-Barough S., Soleimani M. (2017). Fibrin gel as a scaffold for photoreceptor cells differentiation from conjunctiva mesenchymal stem cells in retina tissue engineering. *Artificial Cells, Nanomedicine and Biotechnology*.

[188] Sun W., Motta A., Shi Y. (2016). Co-culture of outgrowth endothelial cells with human mesenchymal stem cells in silk fibroin hydrogels promotes angiogenesis. *Biomedical Materials*.

[189] McCreedy D. A., Wilems T. S., Xu H. (2014). Survival, differentiation, and migration of high-purity mouse embryonic stem cell-derived progenitor motor neurons in fibrin scaffolds after sub-acute spinal cord injury. *Biomaterials Science*.

[190] Zeng X., Zeng Y.-S., Ma Y.-H. (2011). Bone marrow mesenchymal stem cells in a three-dimensional gelatin sponge scaffold attenuate inflammation, promote angiogenesis, and reduce cavity formation in experimental spinal cord injury. *Cell Transplantation*.

[191] Tate C. C., Shear D. A., Tate M. C., Archer D. R., Stein D. G., LaPlaca M. C. (2009). Laminin and fibronectin scaffolds enhance neural stem cell transplantation into the injured brain. *Journal of Tissue Engineering and Regenerative Medicine*.

[192] Gomes E. D., Mendes S. S., Leite-Almeida H. (2016). Combination of a peptide-modified gellan gum hydrogel with cell therapy in a lumbar spinal cord injury animal model. *Biomaterials*.

[193] Lozano R., Stevens L., Thompson B. C. (2015). 3D printing of layered brain-like structures using peptide modified gellan gum substrates. *Biomaterials*.

[194] Lim T. C., Toh W. S., Wang L.-S., Kurisawa M., Spector M. (2012). The effect of injectable gelatin-hydroxyphenylpropionic acid hydrogel matrices on the proliferation, migration, differentiation and oxidative stress resistance of adult neural stem cells. *Biomaterials*.

[195] Du B.-L., Zeng X., Ma Y.-H. (2015). Graft of the gelatin sponge scaffold containing genetically-modified neural stem cells promotes cell differentiation, axon regeneration, and functional recovery in rat with spinal cord transection. *Journal of Biomedical Materials Research Part A*.

[196] Zhang K., Liu Z., Li G. (2014). Electro-acupuncture promotes the survival and differentiation of transplanted bone marrow mesenchymal stem cells pre-induced with neurotrophin-3 and retinoic acid in gelatin sponge scaffold after rat spinal cord transection. *Stem Cell Reviews and Reports*.

[197] Sarnowska A., Jablonska A., Jurga M. (2013). Encapsulation of mesenchymal stem cells by bioscaffolds protects cell survival and attenuates neuroinflammatory reaction in injured brain tissue after transplantation. *Cell Transplantation*.

[198] Xing Q., Zhao F., Chen S., McNamara J., DeCoster M. A., Lvov Y. M. (2010). Porous biocompatible three-dimensional scaffolds of cellulose microfiber/gelatin composites for cell culture. *Acta Biomaterialia*.

[199] Mothe A. J., Tam R. Y., Zahir T., Tator C. H., Shoichet M. S. (2013). Repair of the injured spinal cord by transplantation of neural stem cells in a hyaluronan-based hydrogel. *Biomaterials*.

[200] Schizas N., Rojas R., Kootala S. (2014). Hyaluronic acid-based hydrogel enhances neuronal survival in spinal cord slice cultures from postnatal mice. *Journal of Biomaterials Applications*.

[201] Moshayedi P., Nih L. R., Llorente I. L. (2016). Systematic optimization of an engineered hydrogel allows for selective control of human neural stem cell survival and differentiation after transplantation in the stroke brain. *Biomaterials*.

[202] Nisbet D. R., Moses D., Gengenbach T. R., Forsythe J. S., Finkelstein D. I., Horne M. K. (2009). Enhancing neurite outgrowth from primary neurones and neural stem cells using thermoresponsive hydrogel scaffolds for the repair of spinal cord injury. *Journal of Biomedical Materials Research Part A*.

[203] Nisbet D. R., Rodda A. E., Horne M. K., Forsythe J. S., Finkelstein D. I. (2010). Implantation of functionalized thermally gelling xyloglucan hydrogel within the brain: associated neurite infiltration and inflammatory response. *Tissue Engineering Part: A*.

[204] Wang J., Zheng J., Zheng Q. (2015). FGL-functionalized self-assembling nanofiber hydrogel as a scaffold for spinal cord-derived neural stem cells. *Materials Science and Engineering C: Materials for Biological Applications*.

[205] Horne M. K., Nisbet D. R., Forsythe J. S., Parish C. L. (2010). Three-dimensional nanofibrous scaffolds incorporating immobilized BDNF promote proliferation and differentiation of cortical neural stem cells. *Stem Cells and Development*.

[206] Thouas G. A., Contreras K. G., Bernard C. C. Biomaterials for spinal cord regeneration: outgrowth of presumptive neuronal precursors on electrospun poly(epsilon)-caprolactone scaffolds microlayered with alternating polyelectrolytes.

[207] Havasi P. (2014). The proliferation study of hips cell-derived neuronal progenitors on poly-caprolactone scaffold. *Basic and Clinical Neuroscience*.

[208] Rooney G. E., Knight A. M., Madigan N. N. (2011). Sustained delivery of dibutyryl cyclic adenosine monophosphate to the transected spinal cord via oligo [(polyethylene glycol) fumarate] hydrogels. *Tissue Engineering Part: A*.

[209] Krsko P., McCann T. E., Thach T.-T., Laabs T. L., Geller H. M., Libera M. R. (2009). Length-scale mediated adhesion and directed growth of neural cells by surface-patterned poly(ethylene glycol) hydrogels. *Biomaterials*.

[210] Lampe K. J., Kern D. S., Mahoney M. J., Bjugstad K. B. (2011). The administration of BDNF and GDNF to the brain via PLGA microparticles patterned within a degradable PEG-based hydrogel: Protein distribution and the glial response. *Journal of Biomedical Materials Research Part A*.

[211] Bjugstad K. B., Lampe K., Kern D. S., Mahoney M. (2010). Biocompatibility of poly(ethylene glycol)-based hydrogels in the brain: an analysis of the glial response across space and time. *Journal of Biomedical Materials Research Part A*.

[212] Hakim J. S., Esmaeili Rad M., Grahn P. J. (2015). Positively charged oligo[poly(ethylene glycol) fumarate] scaffold implantation results in a permissive lesion environment after spinal cord injury in rat. *Tissue Engineering Part: A*.

[213] Li H. Y., Führmann T., Zhou Y., Dalton P. D. (2013). Host reaction to poly(2-hydroxyethyl methacrylate) scaffolds in a small spinal cord injury model. *Journal of Materials Science: Materials in Medicine*.

[214] Jhaveri S. J., Hynd M. R., Dowell-Mesfin N., Turner J. N., Shain W., Ober C. K. (2009). Release of nerve growth factor from HEMA hydrogel-coated substrates and its effect on the differentiation of neural cells. *Biomacromolecules*.

[215] Woerly S., Petrov P., Syková E., Roitbak T., Simonová Z., Harvey A. R. (1999). Neural tissue formation within porous hydrogels implanted in brain and spinal cord lesions: ultrastructural, immunohistochemical, and diffusion studies. *Tissue Engineering Part A*.

[216] Plant G. W., Woerly S., Harvey A. R. (1997). Hydrogels containing peptide or aminosugar sequences implanted into the rat brain: Influence on cellular migration and axonal growth. *Experimental Neurology*.

[217] Liu C., Huang Y., Pang M. (2015). Tissue-engineered regeneration of completely transected spinal cord using induced neural stem cells and gelatin-electrospun poly (lactide-co-glycolide)/polyethylene glycol scaffolds. *PLoS ONE*.

[218] Kang K. N., Lee J. Y., Kim D. Y. (2011). Regeneration of completely transected spinal cord using scaffold of poly(D,l-lactide-co-glycolide)/small intestinal submucosa seeded with rat bone marrow stem cells. *Tissue Engineering Part A*.

[219] Teng Y. D., Lavik E. B., Qu X. L. (2002). Functional recovery following traumatic spinal cord injury mediated by a unique polymer scaffold seeded with neural stem cells. *Proceedings of the National Acadamy of Sciences of the United States of America*.

[220] Álvarez Z., Castaño O., Castells A. A. (2014). Neurogenesis and vascularization of the damaged brain using a lactate-releasing biomimetic scaffold. *Biomaterials*.

[221] Hsieh F.-Y., Lin H.-H., Hsu S.-H. (2015). 3D bioprinting of neural stem cell-laden thermoresponsive biodegradable polyurethane hydrogel and potential in central nervous system repair. *Biomaterials*.

[222] Hejčl A., Růžička J., Kapcalová M. (2013). Adjusting the chemical and physical properties of hydrogels leads to improved stem cell survival and tissue ingrowth in spinal cord injury reconstruction: a comparative study of four methacrylate hydrogels. *Stem Cells and Development*.

[223] Kaneko A., Matsushita A., Sankai Y. (2015). A 3D nanofibrous hydrogel and collagen sponge scaffold promotes locomotor functional recovery, spinal repair, and neuronal regeneration after complete transection of the spinal cord in adult rats. *Biomedical Materials*.

[224] Moradi F., Bahktiari M., Joghataei M. T. (2012). BD PuraMatrix peptide hydrogel as a culture system for human fetal Schwann cells in spinal cord regeneration. *Journal of Neuroscience Research*.

[225] Kaneko A., Sankai Y. (2014). Long-term culture of rat hippocampal neurons at low density in serum-free medium: Combination of the sandwich culture technique with the three-dimensional nanofibrous hydrogel PuraMatrix. *PLoS ONE*.

[226] Duan X., Kang E., Liu C. Y., Ming G., Song H. (2008). Development of neural stem cell in the adult brain. *Current Opinion in Neurobiology*.

[227] English D., Sharma N. K., Sharma K., Anand A. (2013). Neural stem cells—trends and advances. *Journal of Cellular Biochemistry*.

[228] Conti L., Cattaneo E. (2010). Neural stem cell systems: physiological players or in vitro entities?. *Nature Reviews Neuroscience*.

[229] Modrek A. S., Bayin N. S., Placantonakis D. G. (2014). Brain stem cells as the cell of origin in glioma. *World Journal of Stem Cells*.

[230] Ramasamy S., Narayanan G., Sankaran S., Yu Y. H., Ahmed S. (2013). Neural stem cell survival factors. *Archives of Biochemistry and Biophysics*.

[231] Lins L. C., Wianny F., Livi S. (2016). Development of bioresorbable hydrophilic-hydrophobic electrospun scaffolds for neural tissue engineering. *Biomacromolecules*.

[232] Shirian S., Ebrahimi-Barough S., Saberi H. (2016). Comparison of capability of human bone marrow mesenchymal stem cells and endometrial stem cells to differentiate into motor neurons on electrospun poly(*ε*-caprolactone) scaffold. *Molecular Neurobiology*.

[233] Carlson A. L., Bennett N. K., Francis N. L. (2016). Generation and transplantation of reprogrammed human neurons in the brain using 3D microtopographic scaffolds. *Nature Communications*.

[234] Delivopoulos E., Shakesheff K. M., Peto H. Neuralization of mouse embryonic stem cells in alginate hydrogels under retinoic acid and SAG treatment.

[235] Bayat N., Ebrahimi-Barough S., Ardakan M. M. M. (2016). Differentiation of human endometrial stem cells into schwann cells in fibrin hydrogel as 3D culture. *Molecular Neurobiology*.

[236] Li H., Koenig A. M., Sloan P., Leipzig N. D. (2014). In vivo assessment of guided neural stem cell differentiation in growth factor immobilized chitosan-based hydrogel scaffolds. *Biomaterials*.

[237] Wei Z., Zhao J., Chen Y. M., Zhang P., Zhang Q. (2016). Self-healing polysaccharide-based hydrogels as injectable carriers for neural stem cells. *Scientific Reports*.

[238] Führmann T., Tam R. Y., Ballarin B. (2016). Injectable hydrogel promotes early survival of induced pluripotent stem cell-derived oligodendrocytes and attenuates longterm teratoma formation in a spinal cord injury model. *Biomaterials*.

[239] Terraf P., Kouhsari S. M., Ai J., Babaloo H. (2017). Tissue-engineered regeneration of hemisected spinal cord using human endometrial stem cells, poly *ε*-caprolactone scaffolds, and crocin as a neuroprotective agent. *Molecular Neurobiology*.

[240] Kim Y.-C., Kim Y.-H., Kim J.-W., Ha K.-Y. (2016). Transplantation of mesenchymal stem cells for acute spinal cord injury in rats: comparative study between intralesional injection and scaffold based transplantation. *Journal of Korean Medical Science*.

[241] Johnson P. J., Tatara A., McCreedy D. A., Shiu A., Sakiyama-Elbert S. E. (2010). Tissue-engineered fibrin scaffolds containing neural progenitors enhance functional recovery in a subacute model of SCI. *Soft Matter*.

[242] Johnson P. J., Tatara A., Shiu A., Sakiyama-Elbert S. E. (2010). Controlled release of neurotrophin-3 and platelet-derived growth factor from fibrin scaffolds containing neural progenitor cells enhances survival and differentiation into neurons in a subacute model of SCI. *Cell Transplantation*.

[243] Qiu X.-C., Jin H., Zhang R.-Y. (2015). Donor mesenchymal stem cell-derived neural-like cells transdifferentiate into myelin-forming cells and promote axon regeneration in rat spinal cord transection. *Stem Cell Research & Therapy*.

[244] Yang Z., Duan H., Mo L., Qiao H., Li X. (2010). The effect of the dosage of NT-3/chitosan carriers on the proliferation and differentiation of neural stem cells. *Biomaterials*.

[245] Shi W., Huang C. J., Xu X. D. (2016). Transplantation of RADA16-BDNF peptide scaffold with human umbilical cord mesenchymal stem cells forced with CXCR4 and activated astrocytes for repair of traumatic brain injury. *Acta Biomaterialia*.

[246] Cheng T.-Y., Chen M.-H., Chang W.-H., Huang M.-Y., Wang T.-W. (2013). Neural stem cells encapsulated in a functionalized self-assembling peptide hydrogel for brain tissue engineering. *Biomaterials*.

[247] Zhang S., Burda J. E., Anderson M. A. (2015). Thermoresponsive copolypeptide hydrogel vehicles for central nervous system cell delivery. *ACS Biomaterials Science and Engineering*.

[248] Shi W., Nie D., Jin G. (2012). BDNF blended chitosan scaffolds for human umbilical cord MSC transplants in traumatic brain injury therapy. *Biomaterials*.

[249] Hwang D. W., Jin Y., Lee D. H. (2014). In vivo bioluminescence imaging for prolonged survival of transplanted human neural stem cells using 3D biocompatible scaffold in corticectomized rat model. *PLoS ONE*.

[250] Zandén C., Hellström Erkenstam N., Padel T., Wittgenstein J., Liu J., Kuhn H. G. (2014). Stem cell responses to plasma surface modified electrospun polyurethane scaffolds. *Nanomedicine: Nanotechnology, Biology and Medicine*.

